# Mindfulness interventions for craving reduction in substance use disorders and behavioral addictions: systematic review and meta-analysis of randomized controlled trials

**DOI:** 10.1186/s12868-023-00821-4

**Published:** 2023-10-18

**Authors:** Anastasia Demina, Benjamin Petit, Vincent Meille, Benoit Trojak

**Affiliations:** 1 Dijon University Hospital, Bâtiment Marion 14 rue Paul Gaffarel, 21079, BP77908 Dijon Cedex, France; 2 INSERM U1093, CAPS, Université de Bourgogne, UFR STAPS, BP 27877, F-21078 Dijon, France

**Keywords:** Mindfulness, Addiction, Craving, Meta-analysis

## Abstract

**Background:**

High-quality evidence is still required to affirm the efficacy of mindfulness-based interventions (MBIs) in craving reduction. MBIs may be particularly appropriate for this purpose given the neurobiological mechanisms of addiction with automatic behavior in response to the negative affect. In this systematic review and meta-analysis, we aimed to study the efficacy of MBIs in craving reduction and to synthetize the newly published data.

**Methods:**

We searched four databases and three clinical trial registries for randomized controlled trials (RCTs) up to August 2023, including studies with MBIs in all types of substance use disorders or behavioral addictions. We chose as our outcome of interest the change from the baseline of craving measures at posttreatment. Standardized mean difference was used as an effect size estimator.

**Results:**

We included 17 RCTs with 1228 participants. The overall effect size was estimated at -0.70 (95% CI -1.15, -0.26) in favor of MBIs.

**Conclusion:**

Due to the high inconsistency (*I*^*2*^ = 92%), we were unable to conclude that there is a medium to large effect size. Overall risk of bias was high for most studies, and the GRADE approach detected a low quality of evidence. Previous clinical and fundamental research suggest that MBIs have a promising potential in addiction medicine. However, further investigation of whether MBIs effectively reduce craving is needed, and innovative solutions for resolving methodological limitations in MBI research are warranted.

**Trial registration:**

PROSPERO registration ID CRD42020221141.

**Supplementary Information:**

The online version contains supplementary material available at 10.1186/s12868-023-00821-4.

## Background

With the current in-depth understanding of the neurophysiological basis of addiction, the definition of substance use disorder (SUD) is difficult to imagine without considering craving. Craving can be defined as a painful urge to engage in a behavior, and it is a multifaceted and challenging phenomenon with cognitive, affective, motivational, and physiological mechanisms [1]. Given the accumulation of recent scientific literature on the subject, the Diagnostic and Statistical Manual of Mental Disorders, Fifth Edition, (DSM-5) included craving in the criteria for SUD [2]. In SUD, craving is a severity indicator and a relapse predictor [1]. It is largely used in motivational approaches to addiction, and it represents an important therapeutic target for addictolytic and substitution medications [1, 3]. Nonetheless, the therapeutic strategies currently used to counteract craving are insufficient, and it is important to develop new approaches for craving reduction [4].

Mindfulness can be defined as a state of consciousness in which a person is fully focused on their experience of the present moment with non-judgmental curiosity and an open attitude [5, 6]. Trait mindfulness or dispositional mindfulness describes a predisposition to be mindful in everyday life, and state mindfulness is attained during mindfulness meditation practice [7, 8].

In 1979, Jon Kabat-Zinn first integrated mindfulness training as an essential part of a 8-week program for therapeutic outcomes, Mindfulness-Based Stress Reduction (MBSR), which became a “gold-standard” intervention for mindfulness-based interventions (MBIs) [9, 10]. These are typically multi-week group interventions, composed of weekly group practice and daily take-home assignments between sessions [11–13]. In these training programs, there is an emphasis on focused attention practice, during which an individual intentionally pays attention to their breathing, noticing mind-wandering, then gently and non-judgmentally bringing their attention back to the breath. This practice is translated into a distinct pattern of activation in various functional networks of the brain, and it leads to improvements in working memory and attentional allocation [14, 15]. A number of MBIs are currently implemented in different clinical settings, such as MBSR, Mindfulness Based Cognitive Therapy (MBCT), and Mindfulness Oriented Recovery Enhancement (MORE), to name a few [13, 16, 17]. MBIs have been extensively studied in a large number of clinical trials addressing anxiety, depression, and stress reduction [18–20].

While the use of MBIs in addiction medicine is more recent, the preliminary evidence is promising [21, 22]. Furthermore, they may be particularly needed in addiction medicine because of the current lack of anti-craving therapies. Indeed, SUD neurobiology studies identify allostatic adaptations in reward circuitry, coupled with executive disruption, resulting in compulsive, automatized substance use when faced with craving [23–25]. In this perspective, MBIs seem particularly appropriate because they can target automatized consumption behavior in response to the negative affect, maladaptive stress management, and particularly craving management. Thought suppression, compensatory behavior (such as going for a walk when experiencing a craving), and other usual avoidance strategies when faced with cravings are often dysfunctional in the long run [26]. On the contrary, the perspective shift offered by regular mindfulness meditation enables the individuals to engage in the attitude of non-judgmental observation of craving sensations, without immediately urging to react when faced with salient cues [27]. A recent review of experimental trials investigating MBIs effect on craving reduction suggests that this effect is generally mediated by working memory load and craving-related response suppression [3]. Indeed, when faced with craving, engaging in moment-to-moment intentional observation of the present experience could reduce painful craving-related elaboration and the urgency to react [3]. The preliminary efficacy of MBIs on various substance use outcomes suggests a therapeutic promise and a significant effect on potential targets of interest, such as stress, thought suppression, reaction to alcohol cues, attentional bias, and psychological flexibility [11, 17, 28]. In addition, these interventions are safe and well accepted by the patients [29].

To implement these interventions in everyday addiction care, practitioners and stakeholders need robust data confirming MBIs effect on craving reduction. Even if the effects of MBIs on SUD outcomes and targets seem promising, meta-analytic data on craving reduction shows mixed results. A significant small to large effect was reported in meta-analyses by Li et al. (9 studies, d = -0.68 (95% CI [-1.11, -0.25]), *I*^*2*^ 83.8%), Grant et al. (9 studies, d = -0.13, 95% CI -0.19 to -0.08, *I*^*2*^ = 0%), and Cavicchioli et al. (d = -0.90 (-1.04; -0.75), *I*^*2*^ 97.28%), there was no evidence of a significant difference from comparison interventions in the meta-analysis by Maglione et al. [4, 30–32]. Importantly, meta-analyses showing the most important MBI effects on craving also report a very high level of inconsistency measured by *I*^*2*^ statistic, precluding conclusions based on these results [33]. In 2020, Korecki and colleagues presented a narrative systematic review of MBI efficacy in SUD suggesting positive and mixed results [34].

The use of MBIs in addiction medicine being a novel and exciting topic, a significant number of RCTs have been published since. We conducted this meta-analysis in view of the considerable scientific production, and to further investigate MBIs effect on craving.

## Methods

### Search strategy

We conducted our review following a published protocol with PROSPERO registration ID CRD42020221141 [35]. The initial study protocol was written before the completion of the scope searches. We adhered to the Preferred Reporting Items for Systematic Reviews and Meta-analyses (PRISMA) guidelines 2020 [36].

We conducted our literature search in November 2020, with the final search update in August 2023, using PubMed (MEDLINE, PubMed Central), EMBASE, PsycINFO via OVID and the Cochrane Library (CENTRAL, Cochrane clinical answers, Cochrane database of systematic review) databases as well as clinical trials registries (clinical trials.gov, EU clinical trials, WHO International Clinical Trials Registry Platform) in order to ensure the screening of unpublished or ongoing trials.

Our search strategy in PubMed/Medline was (“Mindfulness“[MeSH Terms] OR “Mindfulness“[All Fields]) AND (“Craving“[MeSH Terms] OR “Craving“[All Fields]) with a filter for RCTs. We adapted this strategy for the other databases (cf. Additional file 2, Document A for detailed search equations).

### Study selection, inclusion and exclusion criteria

We included all randomized controlled parallel group trials using multisession MBIs for SUD or behavioral addictions (BA) in residential or outpatient settings, counting craving as a primary or a secondary outcome, using all types of craving scales or questionnaires and with data available at a post-treatment time point. We retained studies published in English and French. Studies using all types of control conditions were included. Unpublished trials, conference abstracts, and thesis papers were also eligible for inclusion.

For comparability, we did not include trials in populations with SUD associated with another condition (post-traumatic stress disorder, depression).

Our initial November 2020 protocol was edited in March 2021 to narrow down the inclusion criteria in order to obtain better comparability. For instance, we chose to use the criterion “Mindfulness as an essential component of the studied intervention” and the duration criterion to select only multisession training programs in which mindfulness skills are progressively introduced in form of practical exercises. Thus, we did not include interventions in which mindfulness was taught as only one of different concepts and skills, such as Acceptance and Commitment therapy (ACT), in which mindfulness contributes to one’s engagement in actions in line with one’s values [37]. We also did not include studies with only one or two meditation sessions without any mindfulness training. For better comparability, we chose to exclude double interventions. For example, we did not include studies of MBIs combined with virtual reality.

We implemented all entries in a reference management software, Zotero [38], and, after supervised automatic double removal, two raters (A.D., B.T.) assessed titles and abstracts for inclusion criteria in a blind mode using Rayyan QCRI [39], a systematic review automation tool. Any disagreement after unblinding was resolved by discussion, and when consensus was not obtained, the third rater (B.P.) resolved conflicts. Full texts and references of included articles were then reimplemented on Rayyan QCRI application for blind full-text assessment by two raters (A.D., B.T.). Then, after discussion, conflicts were resolved by the third rater (B.P.). In case of multiple reports on one study, we only used the data set in which craving measures were reported.

### Data extraction and synthesis

The data extraction form was written by A.D. It included the year of recruitment, the year of publication, study location, study funding, study design, availability of the study protocol, blinding, sample size, population characteristics (gender, diagnosis, consumption status), intervention and control characteristics (type of mindfulness intervention, duration, instructors adherence assessment, presence of homework assignments, type of control intervention), as well as all available outcome data with the type of craving scale or questionnaire, data collection points, and results. When different kinds of control conditions were used, the results from the active control group were extracted [40]. Studies were ordered by year of publication. The extraction form was first pilot tested by two authors with 3 randomly selected studies and then it was applied to the data from the included publications. Data extraction was independently performed by two authors (A.D. and B.T.). Data was extracted directly from the published materials when available. When outcome results with sample sizes at post-treatment were not clearly identifiable, the authors were contacted.

### Data analysis

In every included study, outcomes were expressed as means with standard deviations or standard errors. Standard errors were converted into standard deviations using the formula SD = SE x sqrtN [40]. Given the continuous nature of the outcome and the use of different scales and questionnaires for craving assessment in the included studies, we used the standardized mean difference (SMD) to estimate the effect size. The means, the standard deviations in intervention and control groups at post-treatment, and the number of patients in each group were extracted and implemented into Revman 5.4.1 software [41].

In our analysis, we included the available measured data at post-treatment. We considered the potential impact of the missing data on the results in our evaluation. We did not inflate the sample size of the available data up to the total numbers of randomized participants. We chose the inverse variance method and the random effects model in order to consider the differences between studies suggesting clinical and methodological heterogeneity [42]. We used Cohen’s d to describe the SMD for the effect size measure [43]. Usually it is interpreted as small when d = 0.2, as medium when d = 0.5, and large when d = 0.8 [44].

Higgins et al. developed an approach to describe the heterogeneity of studies through the measure of their inconsistency. We used this approach with the *I*^*2*^ statistic describing “the percentage of total variation across studies that is due to heterogeneity rather than chance” [33]. The *I*^*2*^ values lay between 0 and 100%, and higher values indicate a higher level of heterogeneity.

### Risk of bias and quality of evidence

The bias analysis was performed independently by A.D. and B.T. using a revised Cochrane risk-of-bias tool for randomized trials. The following were evaluated: bias arising from the randomization process, deviations from intended interventions, incomplete outcome data management, measure of the outcome, and selection of the reported result [45]. After defining every domain as being at “high risk”, “low risk” or with “some concerns”, A.D. and B.T. evaluated the overall risk. When evaluations did not match, consensus was obtained through discussion with the third author (B.P.). The results were summarized using the Robvis visualization tool [46].

We explored the quality of evidence using the GRADE approach with the GRADEPro tool [47]. Two authors (A.D. and B.T.) completed the GRADE form in blind mode, and the third author (B.P.) resolved conflicts. The GRADE form evaluates the risk of bias, inconsistency, indirectness, imprecision, publication bias, effect size, confounding factors and dose-effect gradient. This evaluation was done using Grade Handbook criteria [48] (GRADE handbook). We accounted for the effect of the smaller studies compared to the larger studies by analyzing publication bias using a funnel plot (cf. Additional file 1).

## Results

### Study selection

**Fig. 1:**
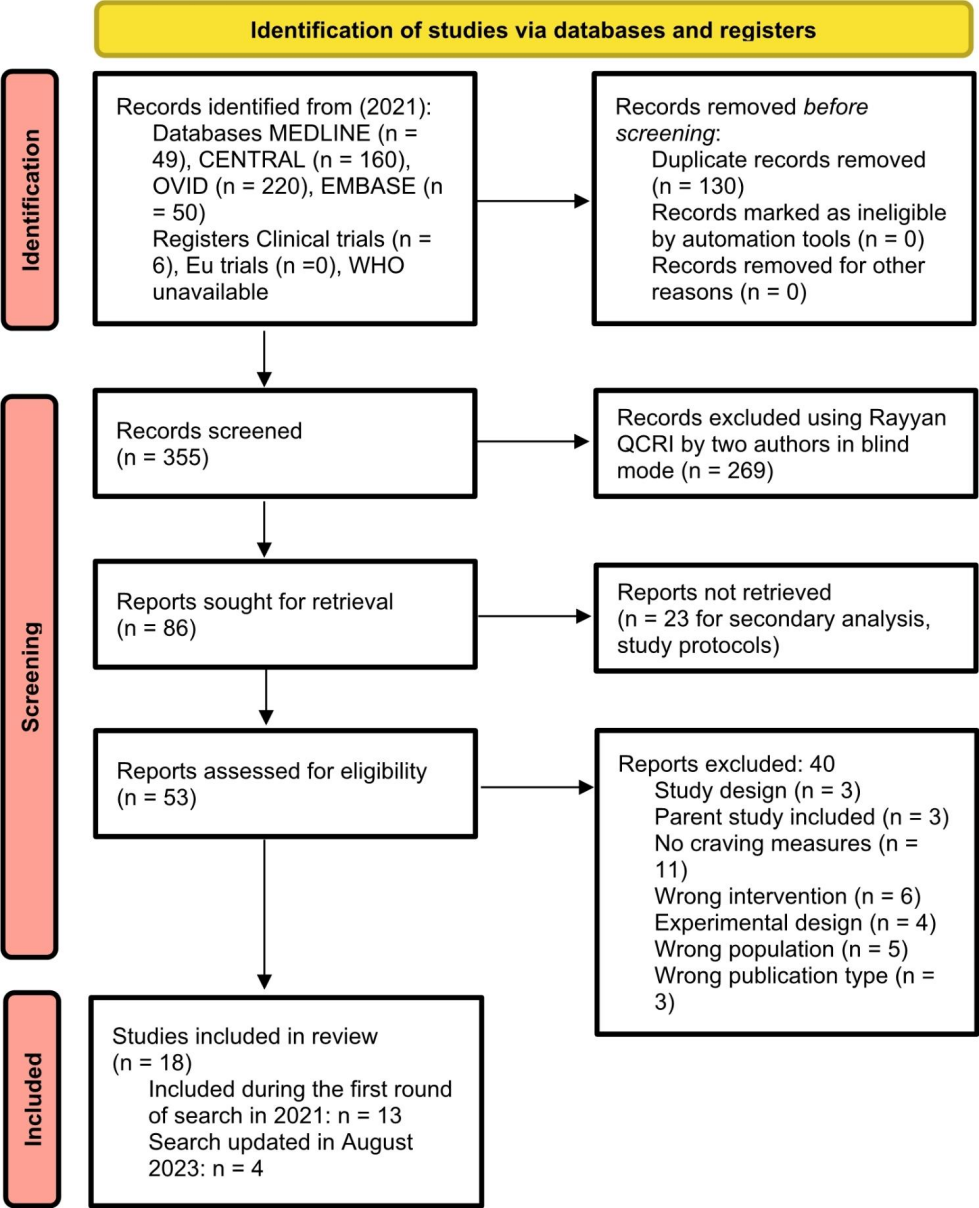
PRISMA 2020 Flow diagram of eligible trials. *From*: Page MJ, McKenzie JE, Bossuyt PM, Boutron I, Hoffmann TC, Mulrow CD, et al. The PRISMA 2020 statement: an updated guideline for reporting systematic reviews. BMJ 2021;372:n71. doi: 10.1136/bmj.n71

Figure 1 shows the detailed selection of included trials (details of excluded trials are available in Additional file 2). Seventeen articles were included, totaling 1228 patients.

Final search round in August 2023 retrieved 11 additional trials, of which 7 were excluded (cf. Additional file 2).

Three corresponding authors of published trials were contacted for precisions about post-treatment outcomes: two authors [49, 50] responded and the data was used in the analysis, one author [51] described a high level of attrition at post-treatment with far less attrition at follow up, so this study was excluded post hoc.

### Characteristics of the studies

The characteristics of the included trials are presented in Table 1. Most trials were recent (with a range of publication years from 2009 to 2023) and more than a half were published between 2018 and 2023. Most trials evaluated patients with SUDs, with only one trial examining the intervention in Internet Gaming Disorder (IGD). In most trials, patients were probably abstaining since they were in the maintenance phase of treatment.

**Table 1:** Characteristics of the included trials

STUDY (author, publication, recruitment, location)	DESIGN (groups, blinding)	SAMPLE SIZE (intervention/control) at randomization = > outcome timepoint	POPULATION (males/females/other, diagnosis, consumption status, residential or outpatient)	INTERVENTION (in patient/app, type, duration, number of sessions, fidelity check, assignments)	CONTROL (type, limitations)	SCALE time point
**Bowen 2009** **USA**^*a*^, **1 center** [17]	RCT^*b*^, 2 parallel groups, no blinding	93/75 = > 62/41	107/61 SUD^*c*^, medically cleared after 2 weeks of treatment, outpatient settings	In patient, MBRP^*d*^, 2 h once a week for 8 weeks (8 sessions), homework assignments (daily exercises on CD^*e*^)	TAU^*f*^ (12 steps based), 1.5 once or twice a week, no homework, no duration matching	PACS^*g*^, Week 8
**Bevan 2010** **USA**^*a*^, **1 center** [21]	RCT^*b*^, 2 parallel groups, researcher blind	35/40 => 35/40	42/33 SUD^*c*^, probably sober (inpatient SUD settings)	In patient, MBSR^*h*^ (5 days Tang protocol) + TAU^*f*^, daily practice during 5 days	TAU^*f*^ + Waitlist, no active control	ACQ-R^*i*^, Week 1
**Garland 2010** **recr**^*i*^**2008, USA**^*a*^, **1 center** [11]	RCT^*b*^, 2 parallel groups, researcher blind	27/26 = > 18/19	42/11 AUD^*j*^, continuous abstinence	In patient, MORE^*k*^, 10 weeks, 1 session a week (10 sessions), homework assignments	ASG^*l*^ (matching themes) ; journaling as homework	PACS^*g*^, Week 10
**Ruscio 2016** **USA**^*a*^, **1 center** [52]	RCT^*b*^, 2 parallel groups, participant blind	24/20 = > 18/14	22/22, people who smoke, outpatient settings	PDA^*m*^ Mindfulness meditation, 2 weeks, daily practice, pre-recorded sessions	Sham meditation	1 to 7 Likert scale, 2 weeks
**Li 2017, recr**^*i*^**2015, USA**^*a*^, **1 center** [59]	RCT^*b*^, 2 parallel groups, dual blinding	15/15 = > 15/14	24/5/1, IGD^*n*^ (subthreshold included)	In patient, MORE^*k*^, 2 h once a week during 8 weeks : 8 sessions, fidelity check, assignments	Support group (active control condition, time matched)	VAS^*o*^ 0–10, 8 weeks
**Shorey 2017, recr**^*i*^**2012–2013, USA**^*a*^**1 center** [28]	RCT^*b*^ 2 parallel groups, no blinding	64/53 = > 62/47	87/30, SUD^*c*^, residential settings, probably sober (28–30 days residential program)	In patient, Residential Mindfulness and Acceptance group therapy, 1.5 h twice a week for 4 weeks (8 sessions) + TAU^*f*^, fidelity check, assignments	TAU^*f*^ (12 steps based, matched on treatment contact time), no structured protocol, no standard group size	PACS^*g*^ adapted (measures for alcohol and drug craving), 4 weeks
**Davis 2018, recr**^*i*^**2015–2016, USA**^*a*^**1 center** [49]	RCT^*b*^, 2 parallel groups, researcher blind	44/35 => 42/31	51/28, SUD^*c*^, probably abstinent (residential treatment)	In patient, MBRP^*d*^ 1.5 h twice a week for 4 weeks (8 sessions) + TAU^*f*^, fidelity check, assignments (20–30 min per day)	Up to 8 AA^*p*^ or NA^*q*^ meetings + TAU^*f*^ (time matched), no assignments	GAIN^*r*^, 4 weeks
**Yaghubi 2018, recr**^*i*^**2017, Iran, multicenter** [60]	RCT^*b*^, 2 parallel groups, no blinding	35/35 = > 35/33	Males with OUD^*s*^, probably abstinent (MMT^*t*^), outpatient settings	In patient, MBRP^*d*^ + MMT^*t*^, 8 weeks, once a week (8 sessions)	MMT^*t*^ + general information (no active control, not matched)	CBQ^*u*^, 8 weeks
**Black 2019, recr**^*i*^**2016–2018, USA**^*a*^**1 center** [61]	RCT^*b*^, 2 parallel groups, Researcher blind	114/111 = > 90/94	Females with SUD^*c*^, probably abstinent, residential settings	In patient, MMWR^*v*^, 80 min twice a week for 6 weeks (12 sessions), fidelity check, assignments (mp3)	NA^*q*^ (time matched), no assignments	PACS^*g*^ modifié, 6 weeks
**Foroushani 2019, Iran, multicenter** [54]	RCT^*b*^, 4 groups (2 experimental and 2 control), no blinding	15/15/15/15 => 13/12/15/15	Males with OUD^*s*^, probably abstinent (MMT^*t*^), outpatient settings	In patient, MBRP^*d*^ + MMT^*t*^, 2 h once a week for 8 weeks, no data on fidelity or assignments	MMT^*t*^ + no intervention, no active control	HCQ^*y*^ (subscales), week 8 (choice of group 1 and 3)
**Price 2019, USA**^*a*^, **3 centers** [62]	RCT^*b*^, 3 conditions (active control choice), no blinding	93/56 = > 74/46	Females with SUD^*c*^, outpatient settings, probably abstinent (after intensive outpatient treatment)	In patient, MABT^*w*^ 1.5 h once a week for 8 weeks, individual (8 sessions) + TAU^*f*^, assignments	TAU^*f*^ + WHE^*x*^ (active control, time matched), take home messages	PACS^*g*^ modified, month 3
**Abed 2019, Iran, multicenter** [53]	RCT^*b*^, 2 parallel groups, no blinding	30/30 = > 24/29	Males with OUD^*s*^, probably abstinent (MMT^*t*^), outpatient settings	In patient, MBRP^*d*^ + MMT^*t*^, 2 h once a week for 8 weeks (8 sessions), assignments	MMT^*t*^ + no intervention, no active control	HCQ^*y*^ (subscales), week 8
**Weiss de Souza 2020, Brazil, 1 center** [63]	RCT^*b*^, 2 parallel groups, no blinding	44/42 = > 12/17	17/69; people who smoke, maintenance treatment, outpatient settings	In patient MBRP^*d*^ 1 h a week for 8 weeks + ST^*z*^, assignments	ST^*z*^ alone, no active control	QSU^*+*^ 1 and 2, 8 weeks
**Skrzynski 2023, USA**^*a*^, **1 center** [50]	RCT^*b*^, 2 parallel groups, no blinding	89/90 => 75/68	94/88; people who reported drinking > 14/21 drinks/week (for females/males, respectively), wishing to reduce or stop their consumption, outpatient settings	In patient MBRP, once a week for 8 weeks	RP, once a week for 8 weeks	AUQ^*++*^ Baseline, mid-treatment and post-treatement
**Harby 2021, Egypt, one center** [64]	RCT^*b*^, 3 parallel groups, open label	20/20/20 = > 15/15/15	Males with OUD^*s*^, abstinent, inpatient treatment	In patient group MBRP^*d*^, 2 h once a week for 8 weeks	CBT– group setting, 2 h once a week for 8 weeks or 12-step program, 90 min once a week	OCDUS^*&*^, DDQ^*&&*^
**Zhang 2022, China, one center** [65]	RCT^*b*^, 2 parallel groups, open label	20/20 = > 19/20	Males with Amphetamine-type stimulant use disorder	10 daily 2-hour class (abbreviated MBRP^*d*^) + TAU^*f*^	10-day TAU^*f*^	VAS^*o*^: 0 (no craving at all) to 10 (extremely intense craving)
**Massaro 2022** [66]	RCT^*b*^, 2 parallel groups, open label	54/54 = > 42/34	92/16, SUD^*c*^, outpatient settings, probably abstinent	In patient individual MBRP, once a week for 8 weeks + TAU CDs for home practice	In patient individual relaxation, once a week for 8 weeks + TAU CDs for home practice	MACS^&&&^

^*a*^USA: United States of America, ^*b*^RCT: Randomized controlled trial, ^*c*^SUD: Substance Use Disorder, ^*d*^MBRP: Mindfulness Based Relapse Prevention, ^*e*^CD: Compact Disk, ^*f*^TAU: Treatment as usual, ^*g*^PACS: Penn Alcohol Craving Scale, ^*h*^MBSR: Mindfulness Based Stress Reduction, ^*i*^ACQ-R: Alcohol Craving Questionnaire revised, ^*j*^recr: recruitement, ^*j*^AUD: Alcohol Use Disorder, ^*k*^MORE: Mindfulness Oriented Recovery Enhancement, ^*l*^ASG: Alcohol Support Group, ^*m*^PDA: Personal Digital Assistant, ^*n*^IGD: Internet Gaming Disorder, ^*o*^VAS: Visual Analog Scale, ^*p*^AA: Alcoholics Anonymous, ^*q*^NA: Narcotics Anonymous, ^*r*^GAIN: Global Appraisal of Individual Needs, ^*s*^OUD: Opioid Use Disorder, ^*t*^MMT: Methadone Maintenance Treatment, ^*u*^CBQ: Craving Beliefs Questionnaire, ^*v*^MMWR: Moment-by-Moment in Women’s Recovery, ^*w*^MABT: Mindful Awareness in Body-oriented Therapy, ^*x*^WHE: Women’s Health Education, ^*y*^HCQ: Heroin Craving Questionnaire, ^*z*^ST: Standard Treatment, ^*+*^QSU: Questionnaire of smoking urges, ^*++*^AUQ Alcohol Urge Questionnaire, ^*+++*^MAT Medication Assisted Treatment, ^*--*^CBT Cognitive Behavioral Therapy, ^*&*^OCDUS Obsessive Compulsive Drug Use Scale, ^*&&*^DDQ Desires for Drug Questionnaire, ^&&&^MACS Multidimentional Alcohol Craving Scale

Mindfulness interventions included the following programs: MBSR, MBRP, MORE, Mindfulness and Acceptance group therapy, Moment-by-Moment in Women’s Recovery, Mindful Awareness in Body-oriented Therapy, as well as mindfulness meditation using a personal digital assistant (PDA). The detailed description of these interventions is available in the Additional file 2. Intervention durations varied from five consecutive days to 12 weeks. Control conditions were sometimes but not always matched on the treatment contact time and theme. Several trials did not have an active control condition using a waitlist, general information sessions, or no intervention. One trial used a sham meditation on a PDA.

The outcome assessments were based on self-reported data using scales (Visual Analog Scale or 1–7 Likert scale) and questionnaires. Detailed description of these scales and questionnaires is available in the Additional file 2. The post-treatment time point depended on the intervention duration and varied from 5 days to 12 weeks.

The included studies were mostly conducted with just the researcher being blinded or in open label conditions. Retention rates depended on study durations and varied from 100% (for a 5-day intervention) to 34% (in the study with 4 weeks of standard treatment and 8 weeks of intervention).

### Effect of mindfulness interventions on craving

**Fig. 2:**
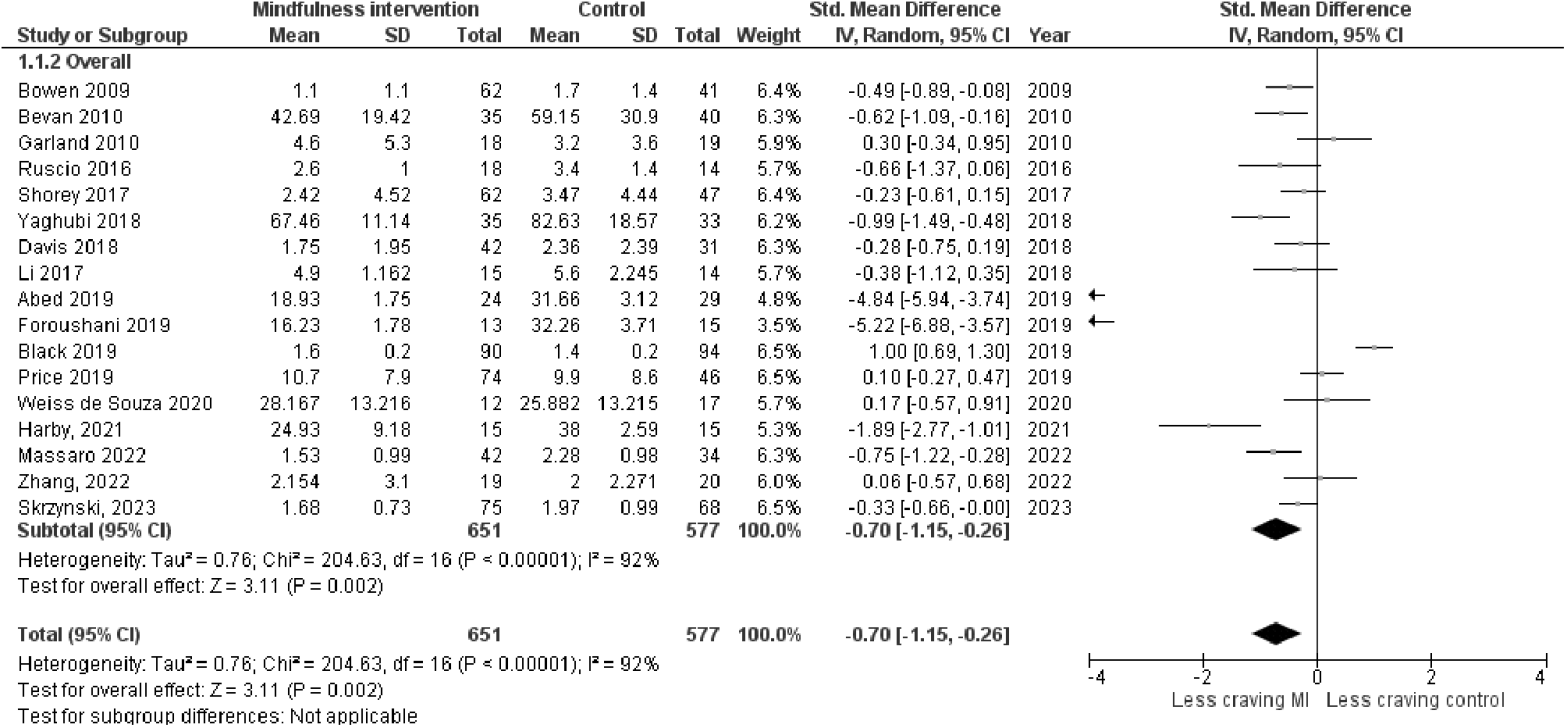
Overall effect of MBIs on craving with effect size, heterogeneity indexes and forest plot

We found an overall significant effect of MBIs on craving reduction, with Cohen’s d at -0.70 (-1.15, -0.26) which could be interpreted as a medium to large effect size (cf. Figure 2 for forest plot). However, like in some of the latest meta-analyses, the *I*² statistic at 92% still indicated a high level of inconsistency between the study results.

We performed a “one study removed” sensitivity analysis and a subgroup sensitivity analysis in an attempt to explain the high degree of heterogeneity between studies. One of the most obvious choices was to perform the analysis without the only trial using a PDA instead of in-patient sessions [52]. The *I²* statistic was not significantly diminished by the removal of this nor any other trial.

We ran our subgroup analyses by population characteristics, taking in account the diagnosis, the sex ratio, the treatment settings, by intervention dose, and by the use of active control. We had 4 condition subgroups: OUD, people who smoke, all SUD and other (one study on IGD and one on AUD). We established 3 sex ratio subgroups on the basis of an arbitrary 70% sex ratio threshold with 3 studies containing > 70% women, 8 studies with > 70% men and 6 studies with both sexes equally present. Six studies explored the effect of MBIs in residential settings and 11 studies in outpatient settings. As for the treatment dose, there were two subgroups with 6 studies on interventions lasting less than 8 weeks of training and 11 studies with those lasting 8 weeks or more. Eleven studies used an active control condition, and 6 studies did not have an active control condition. The results of the subgroup analysis are presented in Table 2 (cf. Additional file 1, Figures A-G for forest plots).

**Table 2:** Results of the subgroup analysis

SUBGROUPS	STUDIES	P VALUE	HETEROGENEITY *(I*^*2*^*)*
Diagnosis: OUD^*g*^/SUD^*a*^/People who smoke/other	4/9/2/2	0.002 / 0.41 / 0.55 / 0.96	95% / 88% / 59% / 47%
Sex ratio: More women/more men/both	3/9/5	0.19 / 0.0009/ < 0.0001	87% / 93% / 0%
Treatment settings: residential/outpatient	6/11	0.43 / 0.001	93% / 91%
Dose: < 8 weeks/ 8 weeks or more	6/11	0.74 / 0.0004	90% / 92%
MBI: MBRP^*b*^/MBSR^*d*^/app/MORE^*e*^/other	5/6/1/2/3	0.006/ 0.002 / 0.07 / 0.96 / 0.44	73% / 95% / NA / 47% / 93%
Control: active/no active control	11/6	0.17 / 0.006	88% / 95%
Blinding: Double/Patient/Researcher/Open label	0/2/4/11	NA/0.05/0.80/0.0002	NA/0%/93%/92%

^*a*^SUD: Substance Use Disorder, ^*b*^MBRP: Mindfulness Based Relapse Prevention, ^*c*^PACS: Penn Alcohol Craving Scale, ^*d*^MBSR: Mindfulness Based Stress Reduction, ^*e*^MORE: Mindfulness Oriented Recovery Enhancement, ^*f*^VAS: Visual Analog Scale, ^*g*^OUD: Opioid Use Disorder, ^*h*^HCQ: Heroin Craving Questionnaire

### Effect by population characteristics

When we ran the subgroup analysis by diagnosis, we found an effect size of -3.14 (-5.13, -1.16) in opioid use disorder (OUD), -0.25 (-1.06, 0.56) in people who smoke and − 0.17 (-0.56, 0.23) in all SUDs. The *I²* statistic was at 95%, 59% and 88%, respectively. There were strong similarities in the OUD studies. Indeed, two of the three studies used the same MBI, had no active control condition, and presented the Heroin Craving Questionnaire (HCQ) by sub-scales. However, the sample sizes in these two studies were slightly different, raising the reasonable question of a center effect.

When we explored the subgroups by sex ratio, the effect size was equal to 0.45 (-0.23, 1.13) in studies with more than 70% women, in those with more than 70% men it was equal to -1.36 (-2.16, -0.56), and when both sexes were represented, Cohen’s d was at -0.43 (-0.63, -0.24), with the *I²* statistic at 87%, 93% and 0%, respectively.

### Effect by treatment

When we ran the subgroup analysis by treatment settings, we found an effect size of -0.28 (-0.97, 0.42) in studies with residential settings and − 0.96 (-1.53, -0.39) in studies with outpatient settings, with the *I²* statistic at 93% and 91%, respectively.

In order to assess whether the number of sessions influenced the outcome, we ran a subgroup analysis by study duration, with an effect size of -0.10 (-0.70, 0.50) for the less-than-8-weeks intervention subgroup and − 1.08 (-1.68, -0.48) for the more-than-8-weeks intervention subgroup. There was a logical significant difference in efficacy between the two subgroups. Nevertheless, the *I²* statistic as high as 90% for the first and 92% for the second subgroup precludes this simple explanation for our main results.

In a subgroup analysis by different mindfulness programs, MBRP study had a Cohen’s d of -0.59 (-1.01, -0.17), studies with MBSR had an effect size of -1.77 (-2.91, -0.63), PDA study had an effect size of -0.66 (-1.37, 0.06), and MORE studies had an effect size of -0.02 (-0.69, 0.66), with an *I*^*2*^ statistic of 73% for MBRP group, 95% for MBSR group, and 47% for MORE.

### Effect of the methodological characteristics of studies

The subgroup analysis by control condition resulted in a Cohen’s d of -0.28 (-0.68, 0.12) in studies with an active control and − 1.74 (-2.97, -0.51) in those without an active control. This logical difference between subgroups did not explain the high level of inconsistency in the main results seeing as the *I²* statistic was 88% for the first subgroup and 95% for the second subgroup.

When we ran the subgroup analysis pooling the studies using a design in which patients were blinded, the effect size was − 0.52 (-1.04, -0.01). In designs in which researchers were blinded, it was 0.11 (-0.72, 0.94). In studies with an open design, d was − 1.06 (-1.62, -0.50). The I² statistic was 0%, 93% and 92%, respectively.

### Risk of bias and quality of evidence

**Fig. 3:**
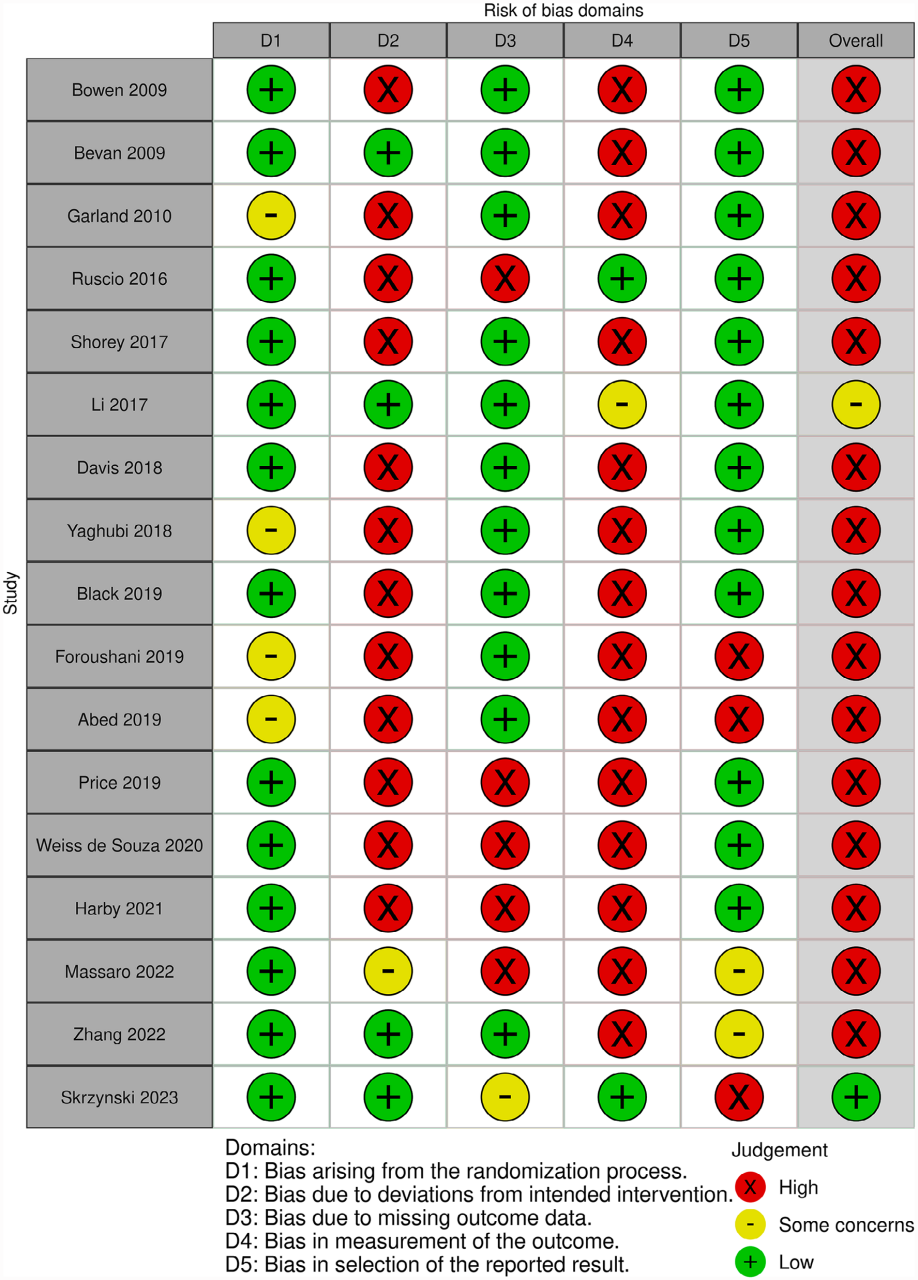
Risk of bias: evaluation of domains from D1 to D5 and overall result. (D1: Bias arising from the randomization process, D2: Bias due to deviations from intended intervention, D3: Bias due to missing outcome data, D4: Bias in measurement of the outcome, D5: Bias in selection of the reported results, X: high risk of bias, - : some concerns, + : low risk of bias)

The results from the Risk of Bias (RoB) evaluation are presented in Fig. 3. The overall RoB was high for the majority of studies, with only two studies [4, 50] with an overall risk of “some concerns”. The D4 evaluation domain (measurement of the outcome) was the most penalizing because patients knew which intervention they were receiving in most trials. The D2 domain (deviations from the intended intervention) was also problematic because of the high attrition rates in the studies. Two studies received a “high risk” evaluation in the D5 domain (selection of the reported results) because of the use of subscales [53, 54].

The GRADE (cf. Additional file 1, Figure I) approach detected a low quality of evidence with serious risk of bias, very serious inconsistency, and serious imprecision with large confidence intervals. No serious publication bias were detected.

## Discussion

The main results of our meta-analysis are encouraging, suggesting a medium-high effect size in favor of the influence of MBIs in craving reduction. However, we found a high degree of inconsistency, making it difficult to draw conclusions relative to this result. We therefore explored our result by performing sub-group analyses which suggested that, despite the use of a random effects model, the observed heterogeneity is the result of various factors, possibly both methodological and clinical. Our review, significantly updating previous systematic reviews, is consistent with their conclusions, reemphasizing the fact that methodological difficulties in MBIs studies continue to be a limiting factor for a reliable quantitative synthesis of all available data.

Craving is a painful and difficult to manage symptom for patients with SUD and BA [26]. The usual distraction or thought repression strategies are not sufficient to cope with this symptom, and sometimes they can even produce its intensification [26, 55]. Mindfulness training offers a different strategy in craving management based on observation and acceptance [17, 23]. Craving is believed to predict substance use and relapse, and a paradigm shift in craving management offered by MBIs could facilitate the effort to obtain sustainable therapeutic outcomes in addiction [56, 57].

Our results underline methodological issues in the included studies. Given that included trials were often underpowered, with high rates of attrition and a high degree of heterogeneity, both methodological and clinical, it is plausible that our results are at least partly mediated by the methodological weaknesses of the included data. In this perspective, continuing investigation of MBI effects on craving intensity and experience is warranted.

Despite the recent proliferation of data regarding MBIs in craving, it may still be difficult to make firm conclusions because of unique methodological issues in mindfulness research. It is difficult to obtain real double blinding, and it is also hard to imagine placebo/control conditions (although sham meditation is sometimes used). In 2015, Davidson proposes a systematic use of an active control condition matched with the time and the length of the intervention. Also a “dual blinding” can be used, in which participants do not know which of the two interventions is experimental [9].

It is also challenging to study craving, partly because it is difficult to establish a solid definition of this critical symptom [58]. In the trials included in this meta-analysis, craving was measured using different scales and questionnaires, possibly contributing to the high degree of heterogeneity between studies. In 2000, Sayette et al. described a variety of ways to measure craving and emphasized that each of these has limitations [55]. The systematic use of simple standardized intensity scales combined with psychometrically validated substance-specific questionnaires could contribute to more comfortable meta-analytic data management. In the included RCTs, craving was sometimes evaluated during the actual craving at a precise point in time and sometimes it was described retrospectively, further adding to the differences between studies and complicating interpretation.

One potential way to overcome some of these challenges would be to conduct trials using digitally assisted mindfulness meditation. A matched active control would be easier to implement in this form, for instance as a mobile phone application with visualization or breathing exercises with the same look and feel as the mindfulness application. With no need for additional human, financial, and infrastructural resources, it can allow larger studies on mindfulness meditation with daily pre-recorded standardized sessions accompanied by key pedagogical messages. Recently, Brewer et al. developed a smartphone application called “Craving to Quit” to be used by smokers using mindfulness meditation for smoking cessation. They found preliminary evidence that the association between craving and smoking was reduced with the use of the application [51]. The use of the ecological momentary assessment approach for real-time craving measures also has a strong potential for craving studies, and it can be easily incorporated in a smartphone application [58].

We recognize that our meta-analysis has limitations. The included trials were often underpowered, with small sample sizes and high rates of attrition. We used measured data in our analysis, which adds to the potential bias in effect size estimations. We chose this option for dealing with missing continuous data, seeing as imputation methods are far from perfect [40]. Craving data is continuous, which adds to the methodological difficulties for missing data, especially in addiction research, where participants are frequently lost to follow-up [17]. Furthermore, it is very likely that the missing data in the included trials was highly dependent on the outcome. Another limitation of this study is the use of the search strategy based on craving. We could have missed trials if the authors did not mention craving evaluation specifically. In addition, we pooled the results from various mindfulness interventions. Although we excluded brief mindfulness training programs for better comparability, there is still a possibility that shorter programs have different impact on clinical outcomes than the longer ones.

Our meta-analysis has strengths: we included recent data with more than half of the studies published after 2015. We conducted our study in accordance with current recommendations for systematic reviews and meta-analyses, aiming for a robust methodology. Even though the main result is comparable to other studies, our inconsistency exploration supports the need for the future developments in mindfulness research recommendations. In fact, a 2021 Cochrane review on MBIs in addiction came to similar conclusions, although craving data was not pooled [6]. Thus, our analysis contributes to the different independent evaluations of MBIs in addiction, revealing inconsistency issues, raising the question of the overly heterogeneous methodological approaches to studying mindfulness interventions.

## Conclusion

Previous fundamental and clinical research suggest that MBIs have a promising potential in addiction medicine. However, the specific effect of MBIs on craving needs to be investigated further, and innovative solutions for designing RCTs in this context are warranted.

### Electronic supplementary material

Below is the link to the electronic supplementary material.

Supplementary Material 1

Supplementary Material 2

## Data Availability

Not applicable.
