# Supplementary material for: Mindfulness interventions for craving reduction in substance use disorders and behavioral addictions: systematic review and meta-analysis of randomized controlled trials
Source: BMC Neurosci. 2023 Oct 18;24:55. doi: 10.1186/s12868-023-00821-4 (PMC10583418; doi:10.1186/s12868-023-00821-4)
Supplement: Supplementary file 1 — Supplementary Material 1 [file 12868_2023_821_MOESM1_ESM.docx]

**ADDITIONAL FILE 2**

**Reasons for exclusion, funding information for the included studies, description of mindfulness-based interventions in included studies, description of craving scales used in included studies, search strategy for all databases and clinical trials registries**

**Table A: Reasons of exclusion (reports assessed for eligibility = 53, 40 excluded, update in 2023 reports assessed for eligibility = 11, 7 excluded)**

| **STUDY** | **REASON OF EXCLUSION** |
| --- | --- |
| Mindfulness as an emerging treatment for smoking and other addictions?  Authors: Brewer, J.;  Journal: Journal of alternative and complementary medicine (New York, N.Y.) - Volume 0, Issue 6, pp. - published 0022-01-01 | Wrong study design |
| Baseline craving strength as a prognostic marker of benefit from smartphone app-based mindfulness training for smoking cessation  2020 APA, all rights reserved)  Authors: Roos, Corey R.; Brewer, Judson A.; O'Malley, Stephanie S.; Garrison, Kathleen A.;  Journal: Mindfulness - Volume 10, Issue 10, pp. 2165-2171 - published 2019-01-01  Publication Types: Journal Article | Parent study already included |
| Surfing the urge: Brief mindfulness-based intervention for college student smokers  Authors: Bowen, Sarah; Marlatt, Alan;  Journal: Psychology of Addictive Behaviors - Volume 23, Issue 4, pp. 666-671 - published 2009-01-01 | Brief intervention |
| Urge surfing as aftercare in adolescent alcohol use:  Authors: Harris, Jennifer S.; Stewart, David G.; Stanton, Brayden C.; Journal: Mindfulness - Volume 8, Issue 1, pp. 144-149 - published 2017-01-01 | Wrong outcome |
| The effects of mindfulness-based yogic breathing on craving, affect, and smoking behavior.  Authors: Lotfalian, Sadaf; Spears, Claire A.; Juliano, Laura M.;  Journal: Psychology of Addictive Behaviors - Volume 34, Issue 2, pp. 351-359 - published 2020-01-01  Publication Types: Journal Article | Wrong intervention |
| Mindfulness-based relapse prevention with individuals receiving medication-assisted outpatient treatment for opioid use disorder  Authors: Zullig, Keith J.; Lander, Laura R.; Sloan, Samantha; Brumage, Michael R.; Hobbs, Gerry R.; Faulkenberry, Laurel;  Journal: Mindfulness - Volume 9, Issue 2, pp. 423-429 - published 2018-01-01  Publication Types: Journal Article | Wrong study design |
| Mindfulness-based relapse prevention for substance use disorders: Effects on cardiac vagal control and craving under stress  Authors: Carroll, Haley; Lustyk, M. Kathleen B.;  Journal: Mindfulness - Volume 9, Issue 2, pp. 488-499 - published 2018-01-01  Publication Types: Journal Article | Wrong outcome |
| Feasibility of a smartphone app with mindfulness training for adolescent smoking cessation: Craving to Quit (C2Q)-teen  Authors: Pbert, Lori; Druker, Susan; Crawford, Sybil; Frisard, Christine; Trivedi, Michelle; Osganian, Stavroula K.; Brewer, Judson;  Journal: Mindfulness - Volume 11, Issue 3, pp. 720-733 - published 2020-01-01 | Wrong outcome |
| Coping with cigarette cravings: Comparison of suppression versus mindfulness-based strategies  Authors: Rogojanski, Jenny; Vettese, Lisa C.; Antony, Martin M.;  Journal: Mindfulness - Volume 2, Issue 1, pp. 14-26 - published 2011-01-01  Publication Types: Journal Article | Wrong intervention |
| Craving and quitting: An exploration of how mindfulness training may help smokers to quit  Authors: Elwafi, Hani M.;  Journal: - Volume 0, Issue 0, pp. - published 2013-01-01  Publication Types: Thesis | Wrong study design |
| Brief psycho-education affects circadian variability in nicotine craving during cessation  Authors: Nosen, Elizabeth; Woody, Sheila R.;  Journal: Drug and Alcohol Dependence - Volume 132, Issue 1, pp. 283-289 - published 2013-01-01  Publication Types: Journal Article | Wrong intervention |
| Mindfulness as a strategy for coping with cue-elicited cravings for alcohol: An experimental examination  Authors: Murphy, Cara M.; MacKillop, James;  Journal: Alcoholism: Clinical and Experimental Research - Volume 38, Issue 4, pp. 1134-1142 - published 2014-01-01  Publication Types: Journal Article Topics: Alcoholics \| Cues | Wrong intervention |
| Mindfulness-Oriented Recovery Enhancement for video game addiction in U.S. emerging adults  Authors: Li, Wen;  Journal: - Volume 0, Issue 0, pp. - published 2017-01-01  Publication Types: Thesis | Parent study already included |
| Effect of mindfulness based relapse prevention on developmental trends, stress, and substance use among young adults in residential substance use treatment: A randomized controlled trial  Authors: Davis, Jordan P.;  Journal: - Volume 0, Issue 0, pp. - published 2019-01-01  Publication Types: Thesis | Parent study already included |
| Effect of a brief meditation intervention on gambling cravings and rates of delay discounting  Authors: Shead, N. Will; Champod, Anne Sophie; MacDonald, Arthur;  Journal: Journal of Mental Health and Addiction - Volume 0, Issue 0, pp. No-Pagination Specified - published 2019-01-01  Publication Types: Journal Article | Wrong population and intervention |
| Using mind control to modify cue-reactivity in AUD: The impact of mindfulness-based relapse prevention on real-time fMRI neurofeedback to modify cue-reactivity in alcohol use disorder: A randomized controlled trial  Authors: Weiss, Franziska; Aslan, Acelya; Zhang, Jingying; Gerchen, Martin Fungisai; Kiefer, Falk; Kirsch, Peter;  Journal: BMC Psychiatry - Volume 0, Issue 0, pp. - published 2020-01-01  Publication Types: Journal Article | Wrong intervention and outcome |
| Mindfulness-based relapse prevention in a jail drug treatment program  Authors: Lyons, Thomas; Womack, Veronica Y.; Cantrell, Wm Dustin; Kenemore, Thomas;  Journal: Substance Use & Misuse - Volume 54, Issue 1, pp. 57-64 - published 2019-01-01  Publication Types: Journal Article | Wrong population |
| The effects of a brief mindfulness exercise on state mindfulness and affective outcomes among adult daily smokers  Authors: Luberto, Christina M.; McLeish, Alison C.;  Journal: Addictive Behaviors - Volume 0, Issue 0, pp. 73-80 – published 2018-01-01  Publication Types: Journal Article | Wrong intervention |
| Improving Functional Outcomes of Veterans With PTSD and Tobacco Dependence  Authors: Development, VA Office of Research and;  Journal: - Volume 0, Issue 0, pp. - published 2020-01-01  Publication Types: BOOK | Wrong population and outcome |
| Moment-by-Moment in Women's Recovery: Randomized controlled trial protocol to test the efficacy of a mindfulness-based intervention on treatment retention and relapse prevention among women in residential treatment for substance use disorder  Authors: Amaro, Hortensia; Black, David S.;  Journal: Contemporary Clinical Trials - Volume 62, Issue 0, pp. 146-152 - published 2017-01-01  Publication Types: Journal Article  Topics: Recurrence \| Substance-Related Disorders | Study protocol |
| Mindfulness Meditation for Health  Authors: Sponsor; University of Wisconsin, Madison; Collaborators; Health (NIH), National Institutes of; Alcoholism (NIAAA), National Institute on Alcohol Abuse and; Party), Information provided by (Responsible; University of Wisconsin, Madison;  Journal: - Volume 0, Issue 0, pp. - published 2010-01-01  Publication Types: BOOK | Study protocol and wrong outcome |
| Role of sensitivity to anxiety symptoms in responsiveness to mindfulness versus  suppression strategies for coping with smoking cravings  Authors: Rogojanski, Jenny; Vettese, Lisa C.; Antony, Martin M.;  Journal: Journal of Clinical Psychology - Volume 67, Issue 4, pp. 439-445 - published 2011-01-01  Publication Types: Journal Article | Wrong outcome |
| Experimental modification of perspective on thoughts and metacognitive beliefs in alcohol use disorder  Authors: Caselli, Gabriele; Gemelli, Antonella; Spada, Marcantonio M.; Wells, Adrian;  Journal: Psychiatry Research - Volume 244, Issue 0, pp. 57-61 - published 2016-01-01  Publication Types: Journal Article | Wrong outcome and intervention |
| Effects of a brief mindfulness-meditation intervention on neural measures of response inhibition in cigarette smokers  Authors: Andreu, Catherine I.; Cosmelli, Diego; Slagter, Heleen A.; Franken, Ingmar H. A.; Verdejo-García, Antonio;  Journal: PLOS ONE - Volume 13, Issue 1, pp. e0191661 - published 2018-01-01  Publication Types: Journal Article | Wrong outcome |
| From the neurobiological basis of comorbid alcohol dependence and depression to psychological treatment strategies: study protocol of a randomized controlled trial  Authors: Becker, Alena; Ehret, Anna M.; Kirsch, Peter;  Journal: BMC psychiatry - Volume 17, Issue 1, pp. 153 - published 2017-01-01 | Wrong population, study protocol |
| Randomized, controlled pilot trial of a smartphone app for smoking cessation using acceptance and commitment therapy  Authors: Bricker, Jonathan B.; Mull, Kristin E.; Kientz, Julie A.; Vilardaga, Roger; Mercer, Laina D.; Akioka, Katrina J.; Heffner, Jaimee L.;  Journal: Drug and Alcohol Dependence - Volume 143, Issue 0, pp. 87-94 - published 2014-01-01  Publication Types: Journal Article | Wrong intervention |
| Immediate effects of interoceptive awareness training through Mindful Awareness in Body-oriented Therapy (MABT) for women in substance use disorder treatment  Authors: Price, Cynthia J.; Thompson, Elaine A.; Crowell, Sheila E.; Pike, Kenneth; Cheng, Sunny C.; Parent, Sara; Hooven, Carole;  Journal: Substance Abuse - Volume 40, Issue 1, pp. 102-115 - published 2019-01-01 | Already included |
| Mindfulness training for smoking cessation: moderation of the relationship between craving and cigarette use  Authors: Elwafi, Hani M.; Witkiewitz, Katie; Mallik, Sarah; Thornhill, Thomas A.; Brewer, Judson A.;  Journal: Drug and Alcohol Dependence - Volume 130, Issue 1, pp. 222-229 - published 2013-01-01  Publication Types: Journal Article | Wrong study design |
| Depression, craving, and substance use following a randomized trial of mindfulness-based relapse prevention  Authors: Witkiewitz, Katie; Bowen, Sarah;  Journal: Journal of Consulting and Clinical Psychology - Volume 78, Issue 3, pp. 362-374 - published 2010-01-01  Publication Types: Journal Article | Parent study already included |
| Mechanisms underlying mindfulness-based addiction treatment versus cognitive behavioral therapy and usual care for smoking cessation.  Authors: Spears, Claire Adams; Hedeker, Donald; Li, Liang; Wu, Cai; Anderson, Natalie K.; Houchins, Sean C.; Vinci, Christine; Hoover, Diana Stewart; Vidrine, Jennifer Irvin; Cinciripini, Paul M.; Waters, Andrew J.; Wetter, David W.;  Journal: Journal of Consulting and Clinical Psychology - Volume 85, Issue 11, pp. 1029-1040 - published 2017-01-01  Publication Types: Journal Article  Topics: Smoke \| Behavior Therapy \| Smoking Cessation \| Cognitive Therapy | Wrong outcome |
| Relative Efficacy of Mindfulness-Based Relapse Prevention, Standard Relapse Prevention, and Treatment as Usual for Substance Use Disorders: A Randomized Clinical Trial  Authors: Bowen, Sarah; Witkiewitz, Katie; Clifasefi, Seema L.; Grow, Joel; Chawla, Neharika; Hsu, Sharon H.; Carroll, Haley A.; Harrop, Erin; Collins, Susan E.; Lustyk, M. Kathleen; Larimer, Mary E.;  Journal: JAMA Psychiatry - Volume 71, Issue 5, pp. 547 - published 2014-01-01  Publication Types: Journal Article | Wrong outcome |
| Therapeutic mechanisms of Mindfulness-Oriented Recovery Enhancement for internet gaming disorder: Reducing craving and addictive behavior by targeting cognitive processes  Authors: Li, Wen; Garland, Eric L.; Howard, Matthew O.;  Journal: Journal of Addictive Diseases - Volume 37, Issue 1, pp. 5-13 - published 2018-01-01  Publication Types: Journal Article  Topics: Internet \| Behavior, Addictive | Parent study already included |
| Effectiveness of mindfulness-based relapse prevention for co-occurring substance use and depression disorders  Authors: Zemestani, Mehdi; Ottaviani, Cristina;  Journal: Mindfulness - Volume 0, Issue 0, pp. No-Pagination Specified - published 2016-01Publication Types: Journal Article | Wrong population |
| Does the MindcotineÂ® virtual reality smartphone app help people to quit smoking?  Authors: ISRCTN50586181;  Journal: http://www.who.int/trialsearch/Trial2.aspx?TrialID=ISRCTN50586181 - Volume 0, Issue 0, pp. - published 2020-01-01  Publication Types: Journal Article  Topics: Smoking \| Smoke | Wrong intervention |
| P05.43 The Effects of a Brief Mindfulness Exercise on State Mindfulness, Smoking, and Affective Outcomes among Adult Smokers  Authors: Luberto Christina (1), McLeish Alison (2)(1) Harvard Medical School / Massachusetts General Hospital; | Wrong intervention |
| Mindfulness-Oriented Recovery Enhancement versus CBT for co-occurring substance dependence, traumatic stress, and psychiatric disorders: Proximal outcomes from a pragmatic randomized trial  Authors: Garland, Eric L.; Roberts-Lewis, Amelia; Tronnier, Christine D.; Graves, Rebecca; Kelley, Karen;  Journal: Behaviour Research and Therapy - Volume 77, Issue 0, pp. 7-16 - published 2016-01-01 | Wrong population |
| Mindfulness-Oriented Recovery Enhancement reduces opioid craving among individuals with opioid use disorder and chronic pain in medication assisted treatment: Ecological momentary assessments from a stage 1 randomized controlled trial  Authors: Garland, Eric L.; Hanley, Adam W.; Kline, Anna; Cooperman, Nina A.;  Journal: Drug and Alcohol Dependence - Volume 203, Issue 0, pp. 61-65 - published 2019-01-01  Publication Types: Journal Article | Wrong population |
| Brief meditation training induces smoking reduction  Authors: Tang, Yi-Yuan; Tang, Rongxiang; Posner, Michael I.;  Journal: Proceedings of the National Academy of Sciences of the United States of America - Volume 110, Issue 34, pp. 13971-13975 - published 2013-01-01  Publication Types: Journal Article | Wrong intervention |
| Enactment of home practice following mindfulness-based relapse prevention and its association with substance-use outcomes  Authors: Grow, Joel C.; Collins, Susan E.; Harrop, Erin N.; Marlatt, G. Alan;  Journal: Addictive Behaviors - Volume 40, Issue 0, pp. 16-20 - published 2015-01-01  Publication Types: Journal Article | Secondary analysis |
| Craving to Quit: A Randomized Controlled Trial of Smartphone App-Based Mindfulness Training for Smoking Cessation  Authors: Garrison, Kathleen A.; Pal, Prasanta; O'Malley, Stephanie S.; Pittman, Brian P.; Gueorguieva, Ralitza; Rojiani, Rahil; Scheinost, Dustin; Dallery, Jesse; Brewer, Judson A.;  Journal: Nicotine & Tobacco Research: Official Journal of the Society for Research on Nicotine and Tobacco - Volume 22, Issue 3, pp. 324-331 - published 2020-01-01 | Excluded after contact with author, data at post-treatment point with high attrition |
| Feasibility and Efficacy of a Brief Mindfulness-Based Smoking Intervention Delivered via the Internet: A Randomized Controlled Trial  Authors : Schmidt MB, Grekin ER, Lumley MA  Journal: Substance use & misuse, 2023, 58(10), 1226‐1234 | Only one meditation session |
| A clinical trial to study the effects of Mindfulness on relapsing factors like craving and emotion regulation, in patients with Alcohol Dependence Syndrome, not yet published  CTRI/2022/03/041523 [Registered on: 31/03/2022] | No answer from the PI |
| Randomized trial of mindfulness- and reappraisal-based regulation of craving training among daily cigarette smokers  Authors: Corey R Roos, Nicholas R Harp, Nilofar Vafaie, Ralitza Gueorguieva, Tami Frankforter, Kathleen M Carroll, Hedy Kober  Journal: Psychol Addict Behav. 2023 Aug 3. | No answer from the corresponding author |
| An Integrated Mechanistic Model of Mindfulness-Oriented Recovery Enhancement for Opioid-Exposed Mother–Infant Dyads  Authors: Sarah E. Reese, Elisabeth Conradt, Michael R. Riquino, Eric L. Garland  Front Psychol. 2021; 12: 688359. | Not an RCT |
| Effectiveness of Mindfulness – Based Therapy and Counseling programs (MBTC) on relapses to methamphetamine dependence at a substance dependency treatment center  Authors: Maneesang W, Hengpraprom S, Kalayasiri R  Journal: Psychiatry research, 2022, 317 | Not an RCT |
| Effectiveness of Mindfulness – Based Therapy and Counseling programs (MBTC) on relapses to methamphetamine dependence at a substance dependency treatment center  Authors: Maneesang W, Hengpraprom S, Kalayasiri R  Journal: Psychiatry research, 2022, 317 | Double |
| Effects of mindful attentional regulation on illicit opioid use for individuals participating in medication assisted treatment: A pilot study. Author: Sooter S  Walden Dissertations and Doctoral Studies | Very high attrition (92% in active group) |
| NCT04233671  Minds and Mentors Program (MiMP)- R61 (MiMP)  Not yet published | Craving data at post-treatment missing |

**Table B: Protocol information and funding**

| **Study** | **Protocole** | **Funding** |
| --- | --- | --- |
| **Bowen 2009** | Formulated hypotheses, no information about pre-registered protocol | National Institute on Drug Abuse grant R21 DA010562 |
| **Bevan 2009** | Formulated hypotheses, no information about pre-registered protocol | No information |
| **Garland 2010** | Formulated hypotheses, no information about pre-registered protocol | Grant Number T32AT003378 from the National Center for Complementary and Alternative Medicine, a  Francisco Varela Research Grant from the Mind & Life Institute, Boulder, CO, and an Armfield-Reeves Innovation Grant from the  UNC School of Social Work, Chapel Hill, NC  Award Number KL2RR025746 from the National Center for Research Resources |
| **Ruscio 2016** | Formulated hypotheses, no information about pre-registered protocol | Uniformed Services University of the Health  Sciences grant number TO72NR awarded to ACR |
| **Li 2017** | Formulated hypotheses, no information about pre-registered protocol | University of North Carolina at Chapel Hill University Research Council Research Grant and Armfield-Reeves Innovation Fund Grant  R01DA042033 |
| **Shorey 2017** | Formulated hypotheses, no information about pre-registered protocol | grants F31AA020131 and K24AA019707 from the National Institute on Alcohol Abuse and Alcoholism (NIAAA)  consulting compensation from Cornerstone of Recovery |
| **Davis 2018** | Pre-registered hypotheses  Davis & Roberts, OSF, 28  June 2017 | National Institute on Drug Abuse (Grant  num.: 1R36DA041538; PI: Davis), the Fahs-Beck Fund for Research and  Experimentation (PI: Davis) (082876), and the Campus Research Board  (Grant num.: RB15434; PI: Roberts) |
| **Yaghubi 2018** | No information | Research Assistant of Isfahan University of Medical Sciences and Health Services (Grant No. 9545) |
| **Black 2019** | Amaro H, Black DS. Moment-by-Moment in Women’s Recovery: Randomized controlled trial protocol to test the efficacy of a mindfulness-based intervention on treatment retention and relapse prevention among women in residential treatment for substance use disorder. Contemp Clin Trials. 2017;62:146–152. | National Institute on Drug Abuse and the National Institute on Alcohol Abuse and Alcoholism (R01DA038648 to D.B. and H.A.) |
| **Foroushani 2019** | No information | Internal funds |
| **Price 2019** | Formulated hypotheses, no information about pre-registered protocol | R01 DA033324 from the National Institute on Drug Abuse, National Institutes of Health |
| **Abed 2019** | No information | No information |
| **Weiss de Souza 2020** | Trial Registration: clinicaltrials.gov.  Identifier: NCT02327104 | Grant number 2013/02316-5 from Fundação de Amparo à Pesquisa do Estado de São Paulo (FAPESP), grant number 870470/1997-3 from Conselho Nacional de Desenvolvimento Científico e Tecnológico (CNPq), grant number APQ 04279-10 from Fundação de Amparo à Pesquisa do Estado de Minas Gerais (FAPEMIG) and grant number 552452/2011 from Coordenação de Aperfeiçoamento de Pessoal de Nível Superior (CAPES) |
| **Skrzynski 2023** | Trial Registration: clinicaltrials.gov.  Identifier: NCT02994043 | R01AA024632 ( U.S. NIH Grant/Contract ) |
| **Harby 2021** | Trial Registration: clinicaltrials.gov.  Identifier: NCT05256485 | No external funding was received by the authors. |
| **Zhang 2022** | No information | National Nature Science Foundation (81871045), Shanghai Clinical Research Center for Mental Health (19MC1911100), Program of Science and Technology Innovation Plan in Shanghai (18411961200), Shanghai Intelligent Engineering Technology Research Center for Addiction and Rehabilitation (19DZ2255200), Shanghai Key Laboratory of Psychotic Disorders (13DZ2260500), Program of Shanghai Science and Technology Committee (19411969200), and Shanghai Mental Health Center (SMHC) Clinical Research Center project (CRC2017YB04). |
| **Massaro 2022** | No information | No information |

**Table C: Mindfulness Based Interventions**

| **INTERVENTION** | **MAIN FEATURES** | **NUMBER AND DURATION OF SESSIONS** | **REFERENCES** |
| --- | --- | --- | --- |
| MBRP  Mindfulness Based Relapse Prevention | Mindfulness based intervention associated with relapse prevention for those in recovery, with training focused on high-risk situations identification, awareness of triggers, coping with negative affect, working with emotions and building a compassionate attitude towards oneself. | 8 weekly 2 hours group sessions with daily individual practice suggested each week. | (Bowen et al., 2009, 2011) |
| 5 days Tang protocol | Intensive training program, inspired by Mindfulness Based Stress Reduction program (8 weeks Mindfulness training program created by Jon Kabat-Zinn) | 5 days, daily training associating formal Mindfulness practice, participants’ feedback. Session duration is 30-45 minutes. | (Kabat-Zinn & Hanh, 2009; Tang et al., 2007) |
| MORE  Mindfulness Oriented Recovery Enhancement | Mindfulness training program inspired by Mindfulness Based Cognitive Therapy (Mindfulness based intervention for depression relapse prevention), with adaptation for patients with Substance use disorders, teaching mindful attitudes towards breathing, walking, and with Mindfulness approach to addiction issues: triggers, craving, at risk situations. | 10 sessions during 10 weeks, with suggested 15 minutes individual practice. | (Garland et al., 2010; Teasdale et al., 2002) |
| PDA Mindfulness  Personal Digital Assistent delivered Mindfulness | Brief Mindfulness intervention: 5 different Mindfulness meditation sessions recorded addressing « urge surfing », breath, body, thoughts, emotions. | 2 weeks, daily practice. | (Ruscio et al., 2016) |
| Residential Mindfulness and Acceptance Therapy | Integration of Mindfulness and Acceptance therapies with open-enrolment group practice in residential settings | Eight 1.5-hour sessions, two sessions per week. Daily exercises for individual practice recorder on CDs. | (Hayes et al., 2004; Shorey et al., 2017) |
| MMWR  Moment-by-Moment in Women’s Recovery | Mindfulness based intervention with focus on relapse prevention and treatment retention in vulnerable women with diverse background and complex history. Adaptation of Mindfulness Based Stress Reduction (cf supra) with an accent on treatment retention in residential settings. | 12 sessions, 2 80 minutes sessions per week. Daily individual practice. | (Black & Amaro, 2019) |
| MABT  Mindfulness Awareness in Body-oriented Therapy | Mindfulness based intervention focusing on bodily sensations, interoceptive awareness | One 90 minutes session per week, for 8 to 10 weeks. Daily individual practice. | (Price et al., 2019) |

Black, D. S., & Amaro, H. (2019). Moment-by-Moment in Women’s Recovery (MMWR): Mindfulness-based intervention effects on residential substance use disorder treatment retention in a randomized controlled trial. Behaviour Research and The- rapy, 120, 103437.
Bowen, S., Chawla, N., Collins, S. E., Witkiewitz, K., Hsu, S., Grow, J., et al. (2009). Mindfulness-based relapse prevention for substance use disorders: a pilot efficacy trial. Substance Abuse, 30(4), 295–305.
Bowen, S., Chawla, N., Grow, J. C., & Marlatt, G. A. (2021). Mindfulness-based relapse prevention for addictive behaviors: a clinician’s guide (Second edition.). New York: The Guilford Press.
Garland, E. L., Gaylord, S. A., Boettiger, C. A., & Howard, M. O. (2010). Mindfulness training modifies cognitive, affective, and physiological mechanisms implicated in alcohol dependence: Results of a randomized controlled pilot trial. Journal of Psychoactive Drugs, 42(2), 177–192.
Hayes, S. C., Strosahl, K., & Wilson, K. G. (2016). Acceptance and commitment therapy: the process and practice of mindful change.
Kabat-Zinn, J., & Hanh, T. N. (2009). Full Catastrophe Living: Using the Wisdom of Your Body and Mind to Face Stress, Pain, and Illness. Random House Publishing Group.
Price, C. J., Thompson, E. A., Crowell, S. E., Pike, K., Cheng, S. C., Parent, S., & Hooven, C. (2019). Immediate effects of interoceptive awareness training through Mindful Awareness in Body-oriented Therapy (MABT) for women in substance use disorder treatment. Substance Abuse, 40(1), 102–115.
Ruscio, A. C., Muench, C., Brede, E., & Waters, A. J. (2016). Effect of Brief Mindfulness Practice on Self-Reported Affect, Craving, and Smoking: A Pilot Randomized Controlled Trial Using Ecological Momentary Assessment. Nicotine & Tobacco Research: Official Journal of the Society for Research on Nicotine and Tobacco, 18(1), 64–73.
Shorey, R. C., Elmquist, J., Gawrysiak, M. J., Strauss, C., Haynes, E., Anderson, S., & Stuart, G. L. (2017). A Randomized Controlled Trial of a Mindfulness and Acceptance Group Therapy for Residential Substance Use Patients. Substance Use & Misuse, 52(11), 1400–1410.
Tang, Y.-Y., Ma, Y., Wang, J., Fan, Y., Feng, S., Lu, Q., et al. (2007). Short-term meditation training improves attention and self-regulation. Proceedings of the National Academy of Sciences, 104(43), 17152–17156.
Teasdale, J. D., Moore, R. G., Hayhurst, H., Pope, M., Williams, S., & Segal, Z. V. (2002). Metacognitive awareness and pre- vention of relapse in depression: Empirical evidence. Journal of Consulting and Clinical Psychology, 70(2), 275–287.

**Table D: Craving scales**

| SCALE | MAIN FEATURES | VALIDITY | REFERENCES |
| --- | --- | --- | --- |
| PACS  Penn Alcohol Craving Scale | Self-report, 5 items with 0 to 6 Likert scales, examining frequency, intensity and duration of craving, ability to resist using and overall impression for the past week. | Yes in Alcohol Use Disorder | (Flannery et al., 1999) |
| ACQ-R  Alcohol Craving Questionnaire Revised | Self-report, 30 items with 1 to 7 Likert scales, evaluation of the craving in the present moment, | Yes in Alcohol Use Disorder | (Raabe et al., 2005) |
| 1-7 Likert scale | Self-report, 1 (Strongly disagree) to 7 (Strongly agree) on « I have strong urges to smoke » | No information | (Ruscio et al., 2016) |
| Visual Analog Scale | Self-report evaluation of current craving experience from 1 (Not at all) to 10 (Extremely) | Yes, in Cocaine Use Disorder and smokers | (Li et al., 2018) |
| GAIN  Global Appraisal of Individual Needs | Self-report 14 craving related items from the GAIN assessment tool | Yes | (Davis et al., 2018; Dennis et al., 2008) |
| CBQ  Craving Beliefs Questionnaire | Self-report evaluation of distorted thoughts in Substance Use Disorders. 20 items with 1 to 7 Likert scale. | Yes | (Küçükkarapınar et al., 2018) |
| HCQ  Heroin Craving Questionnaire | Self-report 45 items with 1 to 7 Likert scale evaluation with 5 sub-scales exploring desire to use, intention to use, anticipation of positive outcome after use, anticipation of relief after use, lack of control over use. | Yes in Heroin Use Disorder | (Tiffany et al., 1995) |
| QSU  Questionnaire of Smoking Urges | Self-report 32 items with 1 to 7 Likert scale evaluation of desire to smoke, anticipation of immediate positive outcome and relief, intention to smoke. | Yes, in smokers | (Tiffany & Drobes, 1991; Toll et al., 2006) |
| AUQ  Alcohol Urge Questionnaire | Self-report 8 items with 1 to 7 Likert scale evaluation of drinking urges. | Yes | (MacKillop, 2006) |
| OCDUS  Obsessive Compulsive Drug Use Scale | Self-report 13-item questionnaire, derived from Obsessive Compulsive Drinking Scale, measuring craving. | Yes, for opioids | (Franken et al., 2002) |
| DDQ  Desires for Drug Questionnaire | Self-report 14-item questionnaire measuring craving. | Yes, for opioids | (Franken et al., 2002) |
| MACS  Multidimensional Alcohol Craving Scale | Self-report 12-item questionnaire measuring desire to consume and loss of control over consumption. | Yes | (Guardia Serecigni et al., 2004) |

Davis, J. P., Berry, D., Dumas, T. M., Ritter, E., Smith, D. C., Menard, C., & Roberts, B. W. (2018). Substance use outcomes for mindfulness based relapse prevention are partially mediated by reductions in stress: Results from a randomized trial. Jour- nal of Substance Abuse Treatment, 91, 37–48.
Dennis, M. L., D, P., D, M. W. P., Titus, J. C., D, P., & S, J. U. M. (2008). GAIN Global Appraisal of Individual Needs: Admi- nistration Guide for the GAIN and Related Measures.

Flannery, B. A., Volpicelli, J. R., & Pettinati, H. M. (1999). Psychometric properties of the Penn Alcohol Craving Scale. Alco- holism, Clinical and Experimental Research, 23(8), 1289–1295.

Franken IHA, Hendriks VM, van den Brink W. Initial validation of two opiate craving questionnaires. Addictive Behaviors. 2002 Sep;27(5):675–85.

Guardia Serecigni J, Segura García L, Gonzalvo Cirac B, Trujols Albet J, Tejero Pociello A, Suárez González A, et al. Estudio de validación de la escala multidimensional de craving de alcohol. Medicina Clínica. 2004 Jul;123(6):211–6.
Küçükkarapınar, M., Eser, H. Y., Kotan, V. O., Yalcinay-Inan, M., Tarhan, R., & Arikan, Z. (2018). Assessing the validity and reliability of the Turkish versions of craving beliefs and beliefs about substance use questionnaire in patients with heroin use disorder: demonstrating valid tools to assess cognition-emotion interplay. Substance Abuse Treatment, Prevention, and Policy, 13(1), 29.

MacKillop J. Factor Structure of the Alcohol Urge Questionnaire Under Neutral Conditions and During a Cue-elicited Urge State. Alcoholism Clin Exp Res. 2006 Aug;30(8):1315–21.

Li, W., Garland, E. L., & Howard, M. O. (2018). Therapeutic mechanisms of Mindfulness-Oriented Recovery Enhancement for internet gaming disorder: Reducing craving and addictive behavior by targeting cognitive processes. Journal of Addictive Diseases, 37(1), 5–13.
Raabe, A., Grüsser, S. M., Wessa, M., Podschus, J., & Flor, H. (2005). The assessment of craving: psychometric properties, factor structure and a revised version of the Alcohol Craving Questionnaire (ACQ). Addiction, 100(2), 227–234.

Ruscio, A. C., Muench, C., Brede, E., & Waters, A. J. (2016). Effect of Brief Mindfulness Practice on Self-Reported Affect, Craving, and Smoking: A Pilot Randomized Controlled Trial Using Ecological Momentary Assessment. Nicotine & Tobacco Research: Official Journal of the Society for Research on Nicotine and Tobacco, 18(1), 64–73.
Tiffany, S. T., & Drobes, D. J. (1991). The development and initial validation of a questionnaire on smoking urges. Addiction, 86(11), 1467–1476.

Tiffany, S. T., Fields, L., Singleton, E., Haertzen, C., & Henningfield, J. E. (1995). The development of a heroin craving questionnaire. Unpublished raw data.
Toll, B. A., Katulak, N. A., & McKee, S. A. (2006). Investigating the factor structure of the Questionnaire on Smoking Urges- Brief (QSU-Brief). Addictive Behaviors, 31(7), 1231–1239.

# Document A: Search strategy

**DATABASE : PUBMED**

Initial search : 19/11/2020, final search 28/08/2023

Search strategy :

("Mindfulness"[MeSH Terms] OR "Mindfulness"[All Fields]) AND ("Craving"[MeSH Terms] OR "Craving"[All Fields])

**DATABASE : THE COCHRANE LIBRARY**

Initial search : 19/11/2020; final search 28/08/2023

Search strategy :

ID Search Hits

#1 ("mindfulness"):ti,ab,kw (Word variations have been searched)

#2 ("craving"):ti,ab,kw (Word variations have been searched)

#3 #2 AND #3

**DATABASE : OVID/PSYCINFO**

Initial search : 20/11/2020; final search 28/08/2023

1. exp Mindfulness-Based Interventions/ OR exp Mindfulness/ or mindfulness.mp OR mindfulness based therapy.mp OR mindfulness meditation.mp OR mindfulness based stress reduction.mp
2. exp Craving/ OR craving.mp OR cravings.mp OR intense desire.mp OR urgent desire.mp OR abnormal desire.mp
3. 1 AND 2

**DATABASE : EMBASE**

Initial search: 20/01/21;

Search strategy :

#1 ('mindfulness' OR 'mindfulness meditation' OR 'mindfulness based stress reduction' OR 'mindfulness based intervention') AND [randomized controlled trial]/lim

#2 'craving' AND [randomized controlled trial]/lim

#3 #1 AND #2

**DATABASE : WHO ICTRP**

Initial search: 20/01/21;

Commentary

Our review was conducted during the COVID-19 outbreak. This particular situation caused heavy traffic generated, leading the database to be temporarily inaccessible from outside WHO. Thus, we could not screen this database, unfortunately.

Final search 28/08/2023:

Mindfulness AND Craving

**DATABASE : CLINICAL TRIALS**

Initial search on 09/02/2021; final search 28/08/2023

Mindfulness AND Craving

**DATABASE : EU TRIALS**

Initial search on 09/02/2021; final search 28/08/2023

Mindfulness AND Craving
