# Supplementary material for: Mindfulness interventions for craving reduction in substance use disorders and behavioral addictions: systematic review and meta-analysis of randomized controlled trials
Source: BMC Neurosci. 2023 Oct 18;24:55. doi: 10.1186/s12868-023-00821-4 (PMC10583418; doi:10.1186/s12868-023-00821-4)
Supplement: Supplementary file 2 — Supplementary Material 2 [file 12868_2023_821_MOESM2_ESM.docx]

**ADDITIONAL FILE 1**

**Forest plots for subgroup analyses, GRADE form and funnel plot**

**Figure A**

**
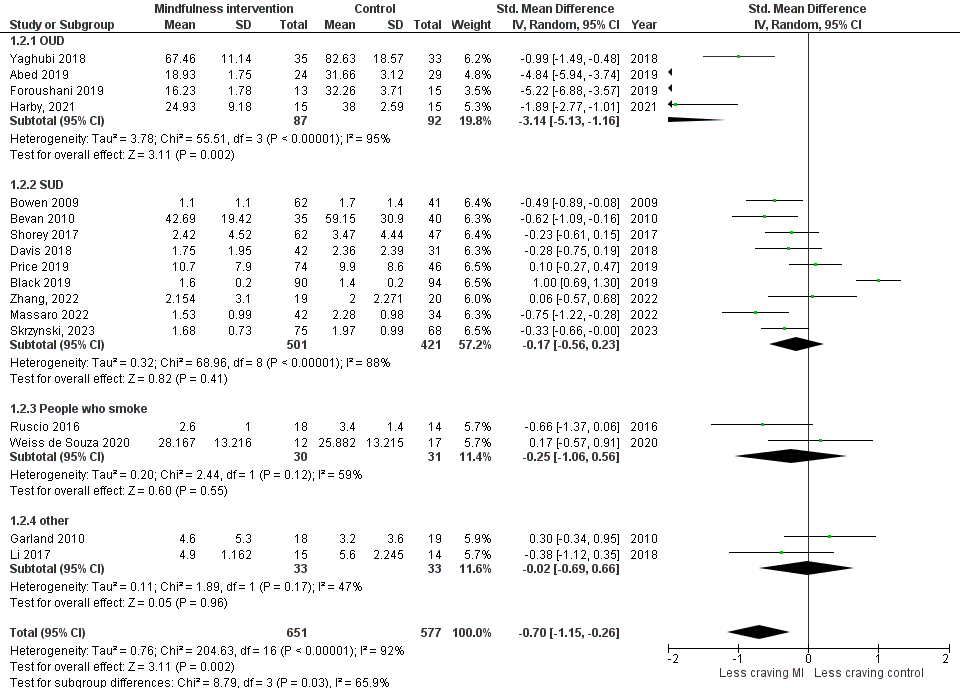
**

**Figure B**


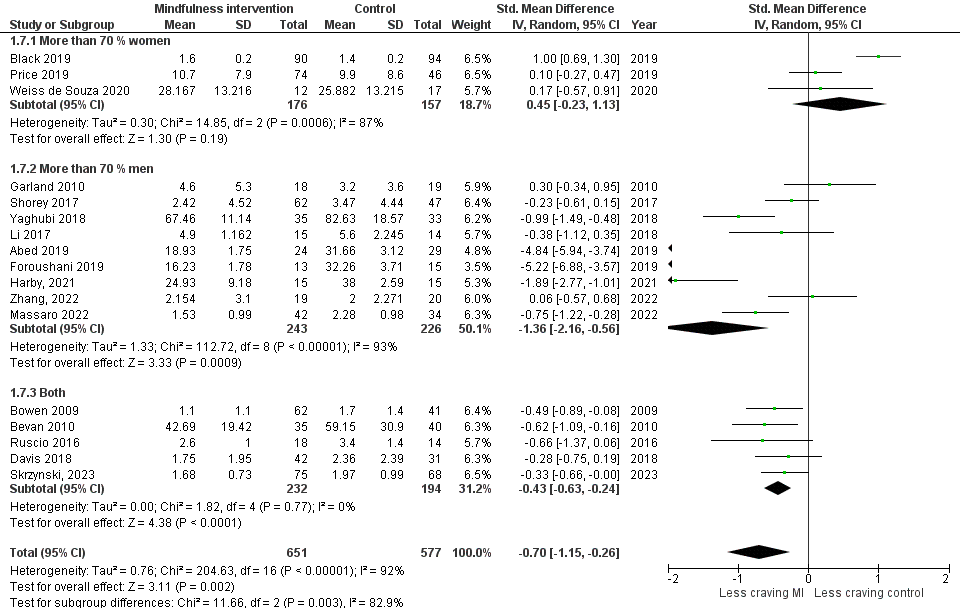


**Figure C**


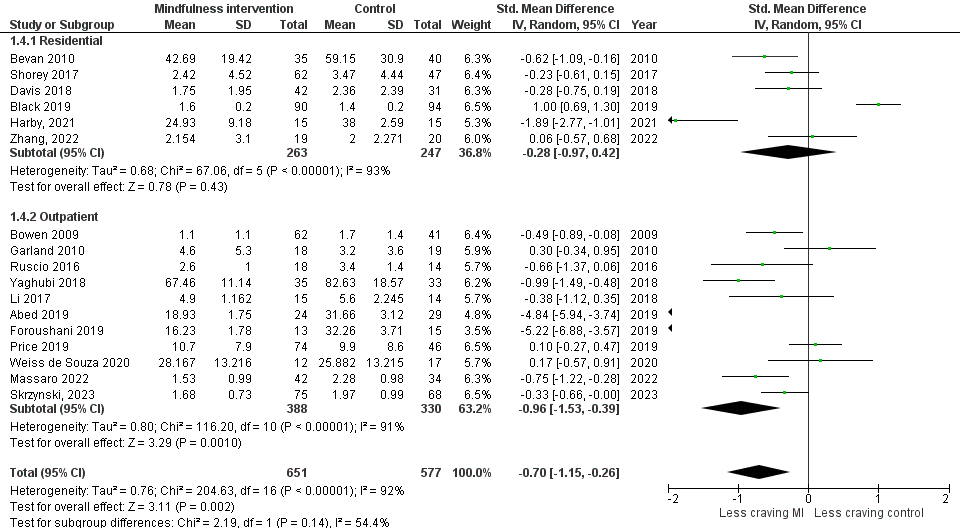


**Figure D**


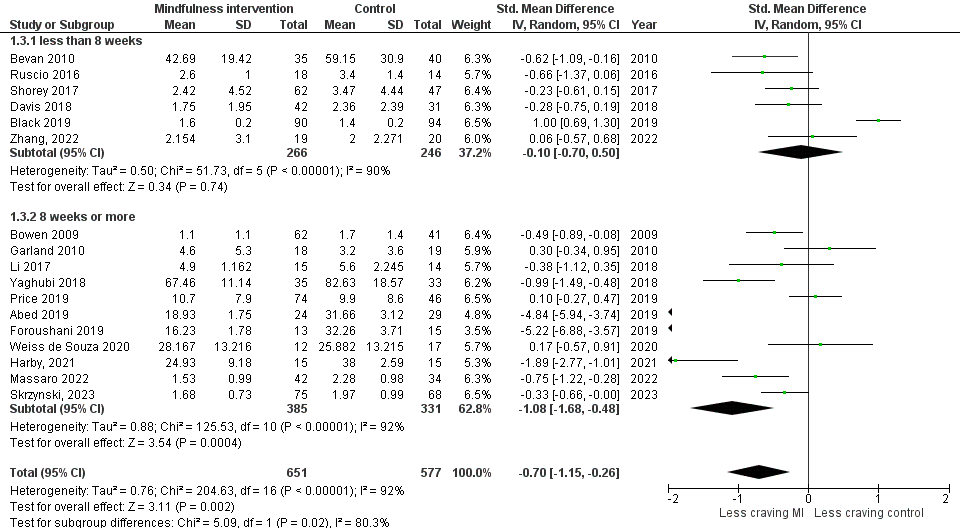


**Figure E**


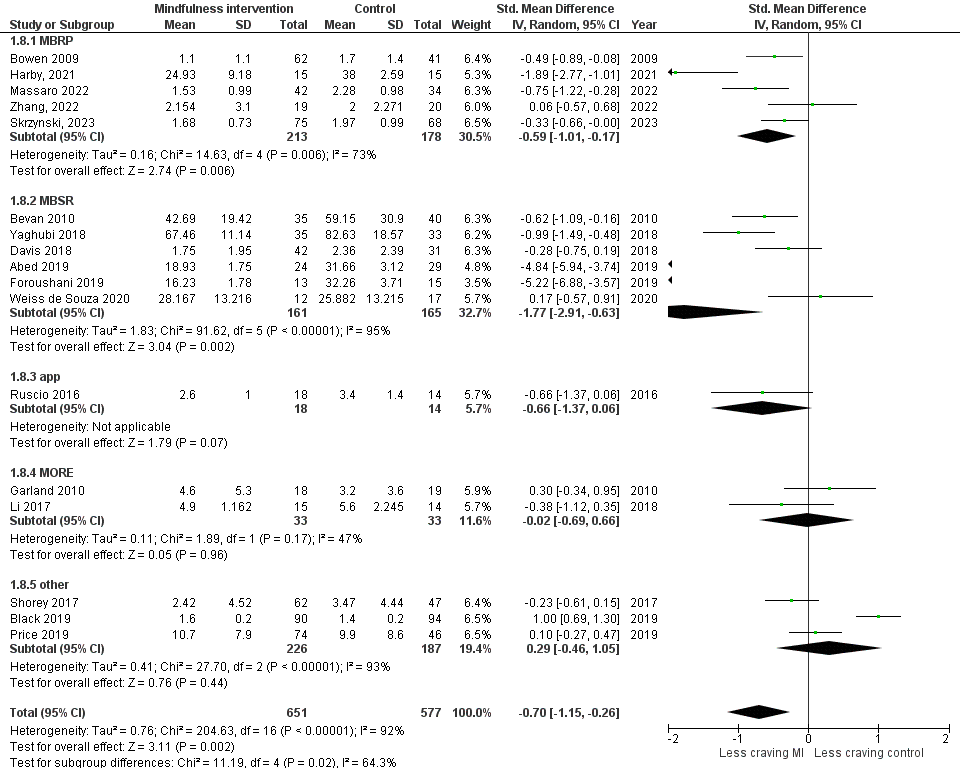


**Figure F**


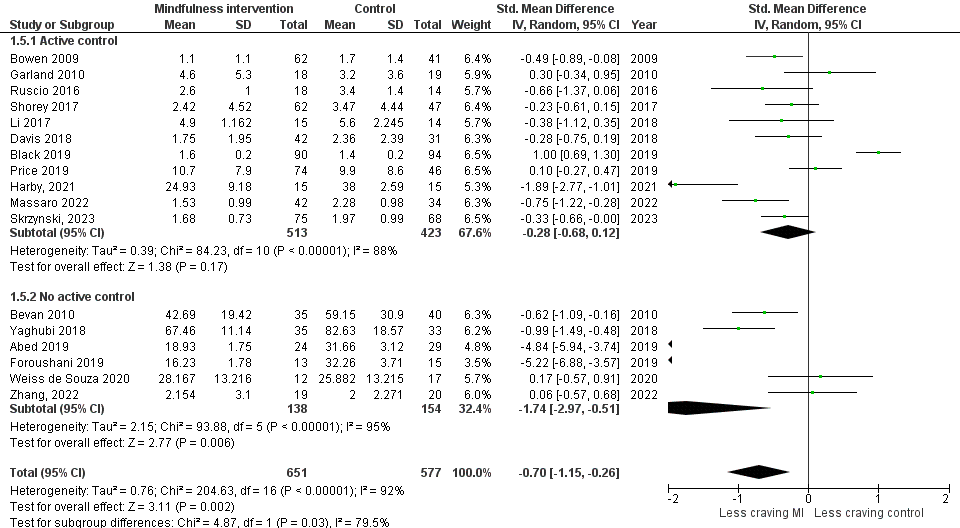


**Figure G**


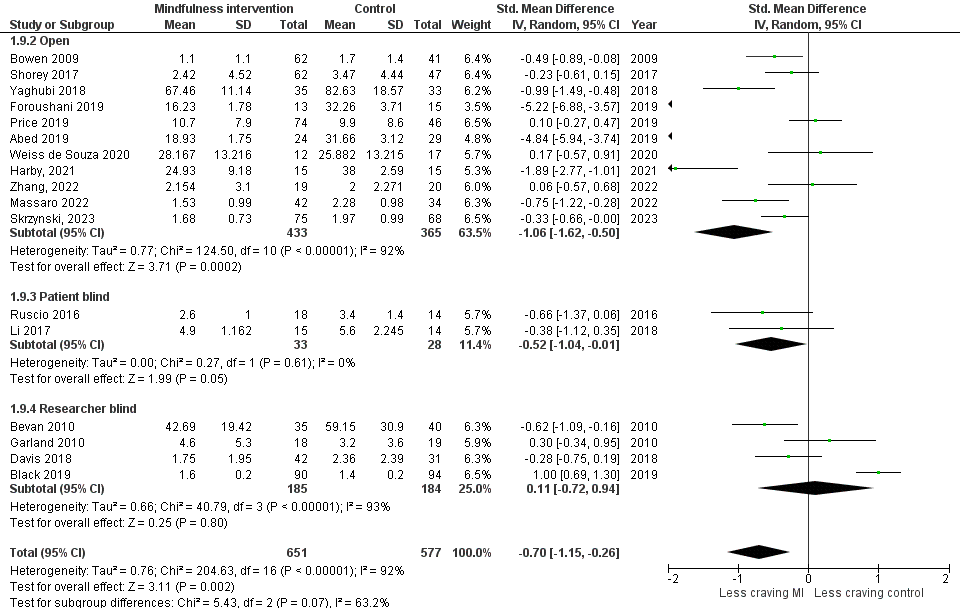


**Figure H**

| **Mindfulness compared to Control for Craving** | | | | | | |
| --- | --- | --- | --- | --- | --- | --- |
| **Certainty assessment** | | | | | | |
| **Participants (studies) Follow-up** | **Risk of bias** | **Inconsistency** | **Indirectness** | **Imprecision** | **Publication bias** | **Overall certainty of evidence** |
|  |  |  |  |  |  |  |
| **Overall craving intensity (assessed with: Craving scales)** | | | | | | |
| 1228 (17 RCTs) | serious^a^ | very serious^b^ | not serious | serious^c^ | strong association dose response gradient | ⨁⨁◯◯ Low |

#### Explanations

a. High attrition, high risk of bias linked to missing data treatment, many studies using self report, overall risk of bias judged high, sometimes there are no active control condition

b. High and unexplained heterogeneity, low overlapping of confidence intervals

c. Large confidence intervals for each study, large final confidence interval

**Figure I**


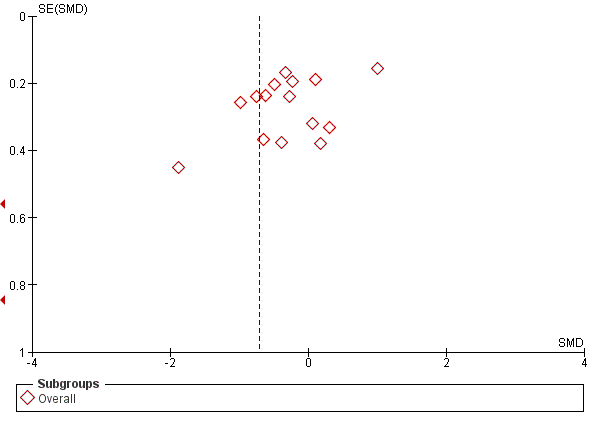


**FIGURE CAPTIONS**

**Figure A:** Subgroup analysis by diagnosis: effect of MBIs on craving with effect size, heterogeneity indexes and forest plot, MBI/MI: Mindfulness-Based Intervention, OUD: Opioid Use Disorder, SUD: Substance Use Disorder, CI: Confidence interval, SD: Standard Deviation, IV: Inverse Variance

**Figure B:** Subgroup analysis by sex ratio: effect of MBIs on craving with effect size, heterogeneity indexes and forest plot MBI/MI: Mindfulness-Based Intervention, CI: Confidence interval, SD: Standard Deviation, IV: Inverse Variance

**Figure C:** Subgroup analysis by treatment settings: effect of MBIs on craving with effect size, heterogeneity indexes and forest plot MBI/MI: Mindfulness-Based Intervention, CI: Confidence interval, SD: Standard Deviation, IV: Inverse Variance

**Figure D:** Subgroup analysis by treatment dose: effect of MBIs on craving with effect size, heterogeneity indexes and forest plot MBI/MI: Mindfulness-Based Intervention, CI: Confidence interval, SD: Standard Deviation, IV: Inverse Variance

**Figure E:** Subgroup analysis by intervention type: effect of MBIs on craving with effect size, heterogeneity indexes and forest plot MBI/MI: Mindfulness-Based Intervention, CI: Confidence interval, SD: Standard Deviation, IV: Inverse Variance MBRP: Mindfulness-Based Relapse Prevention, MBSR: Mindfulness-Based Stress Reduction, app: application, MORE: Mindfulness-Oriented Recovery Enhancement

**Figure F:** Subgroup analysis by control condition: effect of MBIs on craving with effect size, heterogeneity indexes and forest plot MBI/MI: Mindfulness-Based Intervention, CI: Confidence interval, SD: Standard Deviation, IV: Inverse Variance

**Figure G:** Subgroup analysis by blinding: effect of MBIs on craving with effect size, heterogeneity indexes and forest plot MBI/MI: Mindfulness-Based Intervention, CI: Confidence interval, SD: Standard Deviation, IV: Inverse Variance

**Figure H:** Subgroup analysis by craving scale: effect of MBIs on craving with effect size, heterogeneity indexes and forest plot, MBI/MI: Mindfulness-Based Intervention, CI: Confidence interval, SD Standard Deviation, IV: Inverse Variance, HCQ: Heroin Craving Questionnaire, PACS: Penn Alcohol Craving Scale

**Figure I:** GRADE evaluation: RCT: Randomized Controlled Trial, CI: Confidence Interval, SMD: Standardized mean difference

**Figure J:** Funnel plot
